# Supplementary material for: Targeting Microglia/Macrophages Notch1 Protects Neurons from Pyroptosis in Ischemic Stroke
Source: Brain Sci. 2023 Nov 29;13(12):1657. doi: 10.3390/brainsci13121657 (PMC10741505; doi:10.3390/brainsci13121657)
Supplement: Supplementary file 1 [file brainsci-13-01657-s001.zip › brainsci-2723163-supplementary.pdf]

Flow immunoassay of myeloid cells

Figure S1

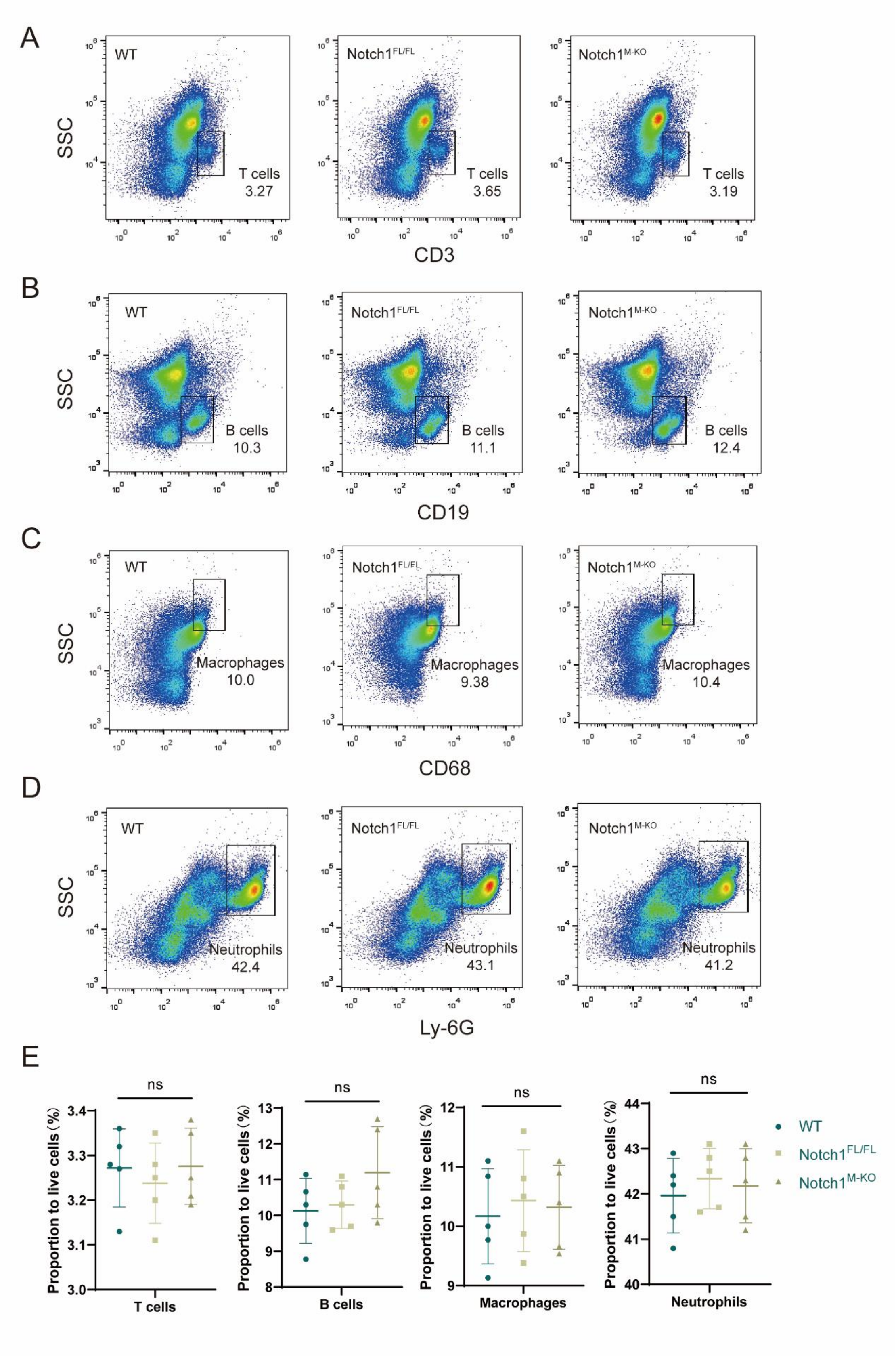

Figure S1

(A) Gating strategy to identify T cells (CD3+). (B) Gating strategy to identify B cells (CD19+). (C) Gating strategy to identify macrophages (CD68+). (D) Gating strategy to identify neutrophils (Ly6G+). (E) Statistical analyses of the proportion of T cells, B cells, macrophages and neutrophils to total live cells (%). (n=5 mice/group), Mean ± SD. ns: no statistical difference.

**Antibody**	**Company**	**Catalogue#**	**Fluorochrome**
CD3	BD Biosciences	561798	FITC
CD19	Biolegend	115528	Alexa Fluor® 700
CD68	Biolegend	137010	PerCP/Cy5.5
Ly6G	BD Biosciences	551461	PE

Figure S2

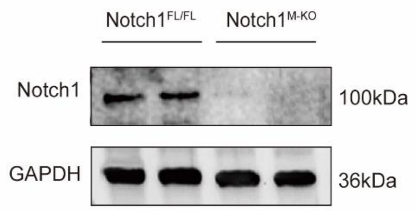

Figure S2

Representative Western blot bands of protein Notch1 in myeloid cells of Notch1$^{M\text{-}KO}$ mice and Notch1$^{FL/FL}$ mice.
